# Pulsed‐Field Ablation for Persistent Atrial Fibrillation in EU‐PORIA Registry

**DOI:** 10.1111/jce.16583

**Published:** 2025-04-02

**Authors:** Jun Hirokami, Kyoung Ryul Julian Chun, Stefano Bordignon, Shota Tohoku, Kars Neven, Tobias Reichlin, Yuri Blaauw, Jim Hansen, Raquel Adelino, Alexandre Ouss, Anna Füting, Laurent Roten, Bart A. Mulder, Martin H. Ruwald, Roberto Mené, Pepijn van der Voort, Nico Reinsch, Thomas Kueffer, Serge Boveda, Elizabeth M. Albrecht, Boris Schmidt

**Affiliations:** ^1^ Cardioangiologisches Centrum Bethanien Frankfurt Germany; ^2^ Department of Electrophysiology Alfried Krupp Hospital Essen Germany; ^3^ Department of Medicine Witten/Herdecke University Witten Germany; ^4^ Inselspital—Bern University Hospital University of Bern Bern Switzerland; ^5^ Department of Cardiology University of Groningen, University Medical Center Groningen Groningen The Netherlands; ^6^ Arrhythmia Unit, Department of Cardiology Gentofte Hospital Copenhagen Denmark; ^7^ Heart Rhythm Department Clinique Pasteur Toulouse France; ^8^ Heart Center Catharina Hospital Eindhoven The Netherlands; ^9^ Boston Scientific Corporation St. Paul Minnesota USA; ^10^ Universitätsklinikum Frankfurt, Medizinische Klinik 3‐ Klinik für Kardiologie Frankfurt Germany

**Keywords:** atrial fibrillation, extra pulmonary vein ablation, persistent AF, propensity score matching, pulmonary vein isolation, pulsed‐field ablation

## Abstract

**Background:**

Real‐life data on efficacy and safety of pulsed‐field ablation (PFA) using the pentaspline multi‐electrode catheter in symptomatic atrial fibrillation (AF) patients is still scarce.

**Objective:**

This study aims to assess the efficacy and safety of PFA in patients with persistent AF.

**Methods:**

Data from early commercial use across seven European centers were collected in a registry. To confirm the efficacy and safety of extra pulmonary vein (PV) ablation, patients were categorized into two groups: those undergoing pulmonary vein isolation (PVI) alone and those receiving additional ablation. Procedural and follow‐up data were collected.

**Results:**

The study included 448 patients (347 PVI only, 101 PVI + *α*). In the PVI + *α* group, extra PV ablation included left atrial posterior wall isolation (87%), mitral isthmus ablation (37%), and cavo‐tricuspid isthmus ablation (3%). At 1‐year follow‐up, the PVI only group showed significantly fewer atrial tachyarrhythmia recurrences compared to PVI + *α* group (69% vs. 56%, *p* = 0.013). While AF recurrence did not significantly differ (25% vs. 28%, *p* = 0.713), PVI + *α* group had a significantly higher atrial tachycardia recurrence (8% *vs.* 22%, *p* < 0.001). Major complications occurred in 2.0% versus 1.0% (PVI only *vs.* PVI + *α*), including pericardial tamponade (6 vs. 0; *p* = 0.345) and stroke (1 vs. 1; *p* = 0.400).

**Conclusions:**

PVI plus extra PV ablation using a pentaspline PFA catheter is associated with a higher incidence of atrial tachycardia recurrences. For persistent AF, a simpler approach of performing only PVI may be more effective.

## Graphical abstract

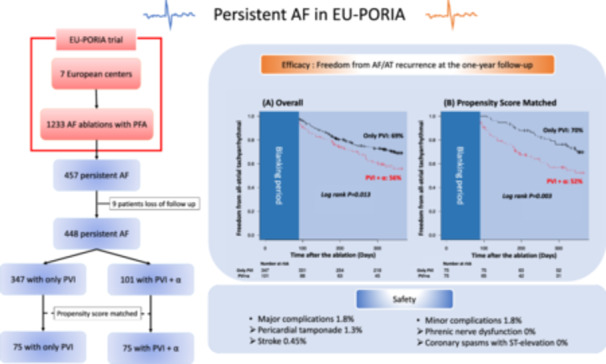

**Abbreviations**
AF: atrial fibrillation
AAD: antiarrhythmic drug
AT: atrial tachycardia
CFAE: complex fractionated electrograms
CTI: cavo‐tricuspid isthmus
LAPW: left atrial posterior wall
MI: mitral isthmus
PFA: pulsed‐field ablation
PV: pulmonary vein
PVI: pulmonary vein isolation

## 1 Introduction

Catheter ablation for paroxysmal atrial fibrillation (AF) is an effective treatment. Triggers of paroxysmal AF mostly come from the pulmonary veins, therefore pulmonary vein isolation (PVI) is an established strategy for AF. However, catheter ablation for persistent AF is more challenging and has a higher recurrence rate of atrial tachyarrhythmia. The STAR‐AF Ⅱ (Substrate and Trigger Ablation for Reduction of Atrial Fibrillation Trial Part II), a multicenter randomized trial, demonstrated that no reduction in the rate of recurrent AF when either linear ablation or ablation of complex fractionated electrograms (CFAE) was performed in addition to PVI [1]. J.A. Clarnette et al also reported that the addition of extra‐pulmonary substrate approaches was associated with declining efficacy when compared to a pulmonary vein ablation alone by conducting a meta‐analysis [2]. On the other hand, the multicenter randomized EARNEST‐PVI (Efficacy of Pulmonary Vein Isolation Alone in Patients With Persistent Atrial Fibrillation) suggested that the PVI alone strategy was non‐inferior to PVI plus linear ablation or CFAE ablation in patients with persistent AF, but implied that the PVI plus strategy was promising to improve the clinical efficacy [3]. Additionally, M. Masuda et al reported that the PVI plus linear ablation was superior to the PVI alone and PVI plus CFAE strategy during extended 3‐year follow up of EARNST‐PVI [4]. The current 2017 h/EHRA/ECAS/APHRS/SOLAECE expert consensus statement describes the complete isolation of the pulmonary veins by linear lesions around their antrum as a cornerstone of AF ablation and recommends to reserve extra pulmonary vein (PV) ablation to select patients only [5].

The durable PVI contributes to be the main factor affecting the AF recurrence rate in patients underwent PVI [6]. Despite the development of ablation technologies and energy sources such as a contact‐force catheter and single‐shot devices, the PV reconnection is frequently observed. Compared to previously described technologies, the durability of PVI using pulsed‐field energy has been contributed to increase lesion durability in a meta‐analysis [7]. Vivek Reddy et al reported that pulsed‐field ablation (PFA) using a pentaspline catheter could safely and effectively treat patients with persistent AF including achieving durable PVI and left atrial posterior wall (LAPW) isolation [8]. The nonthermal PFA ablates selectively atrial myocardium due to a lower threshold for injury than phrenic nerve and esophagus. The pentaspline PFA catheter was developed as a device for PVI, but it holds the potential to safely create durable lesions in extra PV ablation compared to other thermal ablations.

The multi‐center EUropean Real World Outcomes with Pulsed Field AblatiOn in Patients with Symptomatic AtRIAl Fibrillation (EU‐PORIA) registry provide real‐world outcomes from seven high‐volume European AF ablation centers on the early adoption of the novel PFA technology [9]. The trial established high efficacy and safety profile in the ablation for AF using the pentaspline, multi‐electrode PFA catheter. Yet, patients with persistent AF that treated with a pentaspline PFA catheter had significantly a higher recurrence of atrial tachyarrhythmia during follow up compared with paroxysmal AF. In patients with persistent AF registered in the EU‐PORIA registry, a considerable number of individuals underwent extra PV ablation, primarily for substrate modification or in the presence of other coexisting arrhythmias. The objective of this study is to confirm the efficacy and safety of using the pentaspline PFA catheter for extra PV ablation in patients with persistent AF.

### 1.1 Study Design

The EU‐PORIA persistent study was an observational, retrospective evaluation of patients who underwent a PFA for persistent AF during the EU‐PORIA trial to compare arrhythmia recurrence, procedure profiles, and safety outcomes. To confirm the efficacy and safety of extra PV ablation, we conducted an analysis by dividing the patients into two group: one undergoing only PVI and the other undergoing additional extra PV ablation. Seven European high‐volume centers participated in this trial. The trial was approved by each center's ethics committee/institutional review board, registered, and conducted in accordance with the Declaration of Helsinki. Procedural and follow‐up data were collected.

### 1.2 Patients

All patients enrolled in original trial were performed a catheter ablation procedure for symptomatic AF using Farapulse PFA system from 25 March 2021 to 31 May 2022. A total of 457 patients from EU‐PORIA were included in this trial (352 only PVI and 105 PVI + *α*).

### 1.3 Ablation Procedure

The ablation procedure protocol has been previously described in the EU‐PORIA trial [9]. Ablation procedures were conducted in accordance with each center's standard of care. These procedures were carried out under either general anesthesia or deep sedation, employing a continuous infusion of propofol. The guidance for the procedures varied among centers, with some utilizing 3D electroanatomical mapping, while others employed the pentaspline catheter with fluoroscopic guidance alone. The Farawave™ ablation catheter was introduced into the left atrium through a steerable sheath (13.0 F inner diameter; Faradrive™) and navigated over‐the‐wire to the designated ablation area. For ablation, PFA applications were administered using the Farastar™ generator, delivering a voltage output ranging from 1.8 to 2.0 kV. Energy applications were administered as a biphasic waveform on a microsecond scale, unsynchronized with the cardiac rhythm [10]. Each PFA application consisted of a group of five consecutive pulse trains, totaling 2.5 s of ablation time. PFA lesion sets were carried out based on the standard of care at each institution. During conduct this study, the use of the Farapulse PFA System for treating extra‐PV ablation was beyond the labeled indication. When lesion formation was not successful with the pentaspline PFA catheter alone, additional ablation was performed with an irrigated RF catheter.

### 1.4 Repeat Ablation

Patients with symptomatic atrial tachycardia (AT)/AF recurrences underwent repeat mapping and ablation procedures using a 3D mapping system. PVI durability was assessed with multipolar mapping catheters, and the durability of extra‐pulmonary vein ablation lesion sets (such as conduction block of linear lesions or durable posterior wall isolation) was evaluated. Repeat ablation was performed using commercially available irrigated RF ablation catheters.

### 1.5 Outcome Measures

Outpatient visits including 12‐lead ECG or 24–120 h Holter ECG were performed 3‐, 6‐ and 12‐month follow‐up. The primary outcome of the study was any atrial tachyarrhythmia recurrence documented by the ECG tests during the follow‐up period after the index ablation procedure. Atrial tachyarrhythmia was defined as the presence of AF, atrial flutter (AFL), and AT lasting > 30 s detected using 12‐lead ECG or other appropriate tests. The first 3 months after the last ablation was considered as the blanking period.

The secondary outcomes of the study were tamponade, air embolism, stroke, transient ischemic attack, atrio‐esophageal fistula, and death. The relevance of each adverse event to the device and/or procedure was determined by the participating center.

### 1.6 Statistical Analysis

Continuous variables were expressed as mean ± SD and categorical variables as number and percentage. Unpaired Student's *t*‐test, Mann–Whitney U test, or *χ* ^2^ test/Fisher's exact test was used to compare the two groups. We used logistic regression to estimate the propensity score, including the following covariates: age, gender, body mass index, hypertension, diabetes mellitus, heart failure, first AF ablation and prior use of class Ⅰ or Ⅲ antiarrhythmic drug (AAD). Based on their propensity score, patients who underwent only PVI or PVI + *α* were matched on a 1:1 basis with the nearest neighbor algorithm, without replacement, using a caliper width 0.2 logit of the standard deviation. The Kaplan–Meier method was employed to estimate the cumulative incidence and assess potential differences using the log‐rank test. To identify independent predictors of freedom from the primary outcome, Cox regression analysis was conducted in multivariate models. Priori variables were selected based on previous literature and clinical importance. A *p*‐value < 0.05 was considered statistically significant. Statistical analyses were conducted using the R software version 4.2.1 (R Foundation for Statistical Computing, Vienna, Austria).

## 2 Results

### 2.1 Patient Characteristics

Details of the patient characteristics are given in Table 1. In brief, mean age was 68 ± 10 years, and 154/448 (34%) patients were female. The mean CHA2DS2‐VASc score was 2.6 ± 1.6. In 222/448 (50%) patients, ablation was performed with current or previous use of membrane active AAD. In this trial, of the 448 PFA procedures were performed, 425 (95%) were index procedures, and 23 (5%) were repeat procedure after an initial thermal procedure.

**Table 1:** Patient Characteristics.

	Entire cohort			Propensity score matched		
	only PVI	PVI + *α*		only PVI	PVI + *α*	
	*n* = 347	*n* = 101	*p* value	*n* = 75	*n* = 75	*p* value
Age (years)	68 ± 10	68 ± 9	0.657	69 ± 8	68 ± 8	0.331
Height (cm)	176 ± 9	174 ± 11	0.090	174 ± 9	174 ± 11	0.887
Weight (kg)	88 ± 18	87 ± 20	0.734	89 ± 19	87 ± 20	0.455
BMI (kg/m^2^)	28 ± 5	29 ± 6	0.534	29 ± 5	28 ± 5	0.396
Female sex, *n* (%)	115 (33)	39 (39)	0.341	28 (37)	28 (37%)	1.000
Hypertension, *n* (%)	216 (62)	56 (55)	0.247	50 (68)	41 (55)	0.181
Diabetes, *n* (%)	49 (14)	23 (23)	0.045	20 (25)	18 (22)	0.853
History of stroke/TIA, *n* (%)	23 (7)	8 (8)	0.658	6 (8)	7 (9)	1.000
Heart failure, *n* (%)	94 (27)	18 (18)	0.067	18 (24)	17 (23)	1.000
Coronary artery disease, *n* (%)	62 (18)	17 (17)	0.883	17 (23)	13 (17)	0.541
CHA2DS2‐VASc score	2.6 ± 1.6	2.6 ± 1.6	0.997	2.9 ± 1.4	2.6 ± 1.6	0.194
Prior use of Class Ⅰor Ⅲ AAD, *n* (%)	160 (46)	62 (61)	0.009	39 (52)	42 (56)	0.743
Left ventricular ejection fraction (%)	54 ± 11	54 ± 10	0.982	55 ± 11	53 ± 10	0.270
First AF ablation, *n* (%)	346 (99.7)	79 (78)	< 0.001	74 (99)	74 (99)	1.000

*Note*: CHADS2/CHA2DS2‐VASc, congestive heart failure, hypertension, age ≥ 75 years, diabetes mellitus, history of stroke or TIA, vascular disease, age 65–74, and sex category.

Abbreviations: AAD, antiarrhythmic drug; AF, atrial fibrillation; BMI, body mass index; PVI, pulmonary vein isolation; TIA, transient ischemic attack.

**Figure 1:**
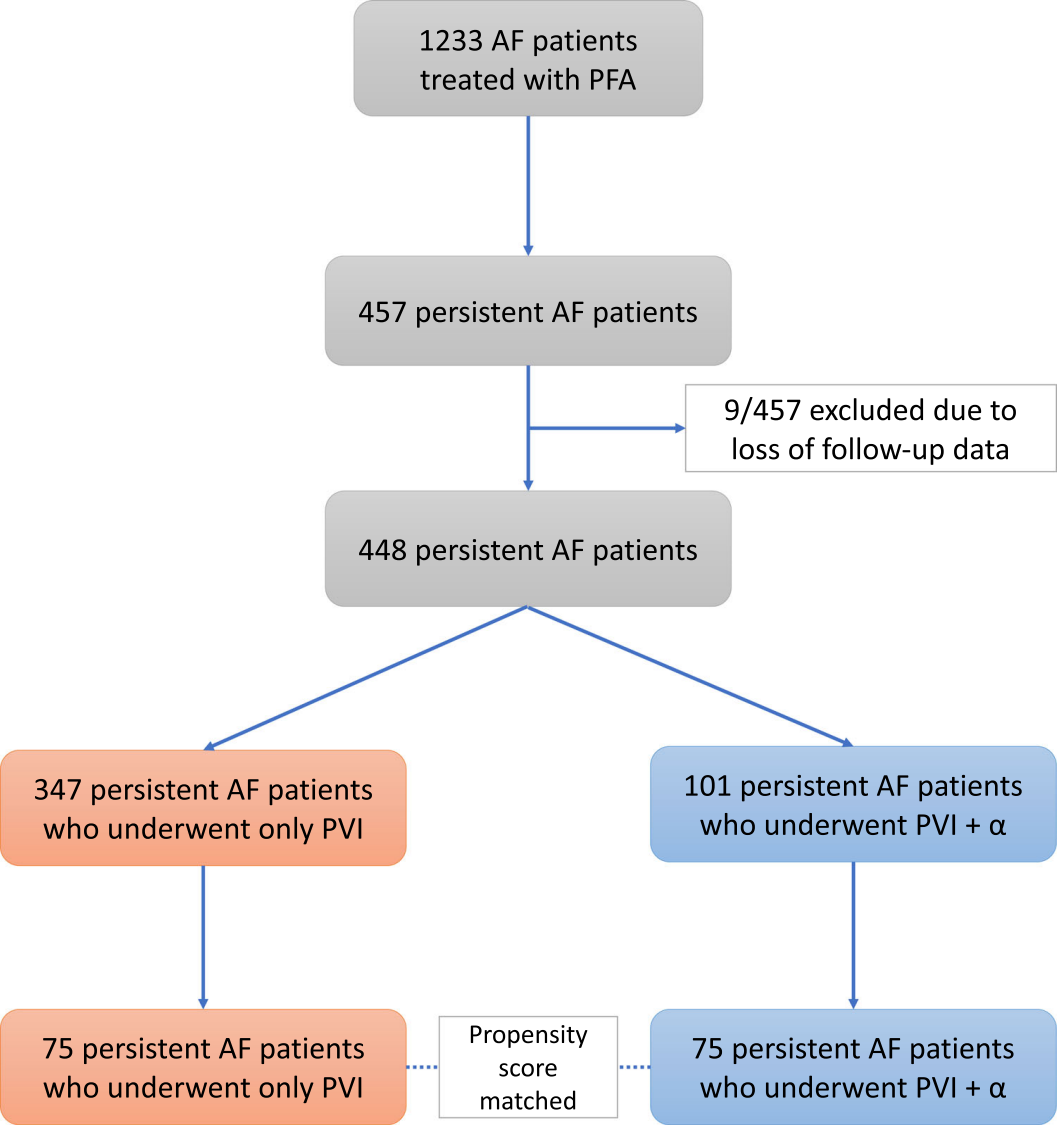
Study flowchart. Abbreviations: AF, atrial fibrillation; PFA, pulsed‐field ablation; PVI, pulmonary vein isolation.

The detailed study flowchart is summarized in Figure 1. A follow‐up analysis was conducted on 448 patients with persistent AF who underwent AF ablation using a pentaspline PFA catheter (only PVI: *n* = 347, PVI + *α*: *n* = 101).

### 2.2 Procedural Characteristics and Ablation Results

Procedural characteristics are summarized in Table 2. In the only PVI group, 1368 PVs were treated, whereas in the PVI+ group, 387 PVs were treated. All PVs were successfully isolated using only PF energy. Among the PVI + *α* cohort, additional extra PV ablation alongside PVI was performed; 88/101 (87%) LAPW isolation, 37/101 (37%) mitral isthmus (MI) ablation and 3/101 (3%) cavo‐tricuspid isthmus (CTI) ablation. Among the extra PV ablations, acute lesion formation failed in 1/88 (1%) lesions for LAPW isolation and in 7/37 (19%) lesions for MI ablation. In those lesions, irrigated RF touch‐up ablation at residual conduction gaps was performed. More ablation procedures in the PVI + *α* cohort were carried out under general anesthesia (13% vs. 49%, *p* < 0.001) and with 3D‐mapping system (38% vs. 59%, *p* < 0.001). The 35 mm pentaspline PFA catheter was more frequently utilized in the PVI + *α* group (26% vs. 51%, *p* < 0.001). The procedural and fluoroscopy times were longer in the PVI + *α* group (67 ± 37 min vs. 102 ± 44 min; *p* < 0.001 and 16 ± 9 min vs. 24 ± 10 min; *p* < 0.001).

**Table 2:** Procedural Characteristics.

	Entire cohort			Propensity score matched		
	only PVI	PVI + *α*		only PVI	PVI + *α*	
	*n* = 347	*n* = 101	*p* value	*n* = 75	*n* = 75	*p* value
Sedation technique						
General anaesthesia, *n* (%)	46 (13)	49 (49)	< 0.001	13 (17)	37 (49)	< 0.001
Deep sedation, *n* (%)	301 (87)	54 (51)		62 (83)	38 (51)	
Use of 3D mapping, *n* (%)	130 (38)	60 (59)	< 0.001	25 (33)	46 (61)	0.001
Skin‐to‐skin procedure time (min)	67 ± 37	102 ± 44	< 0.001	63 ± 34	100 ± 40	< 0.001
Fluoroscopy time (min)	16 ± 9	24 ± 10	< 0.001	16 ± 9	24 ± 10	< 0.001
Ablation device used						
31 mm, *n* (%)	258 (74)	50 (49)	< 0.001	60 (80)	41 (54)	0.001
35 mm, *n* (%)	89 (26)	51 (51)		15 (20)	34 (46)	
Extra‐PV ablation						
Posterior wall isolation, *n* (%)	0 (0)	88 (87)	NA	0 (0)	67 (89)	NA
Mitral isthmus ablation, *n* (%)	0 (0)	37 (37)	NA	0 (0)	28 (37)	NA
Cavo‐tricuspid isthmus ablation, *n* (%)	0 (0)	3 (3)	NA	0 (0)	3 (4)	NA
Type of recurrence						
AF	89 (26)	23 (23)	0.603	18 (24)	20 (27)	0.851
AT/AFL	24 (7)	19 (19)	0.001	5 (7)	15 (20)	0.029

Abbreviations: AF, atrial fibrillation; AFL, atrial flutter; AT, atrial tachycardia; PV, pulmonary vein; PVI, pulmonary vein isolation.

## 3 Efficacy Outcomes

### 3.1 Freedom From All‐Atrial Arrythmia at the 1‐Year Follow‐Up

**Figure 2:**
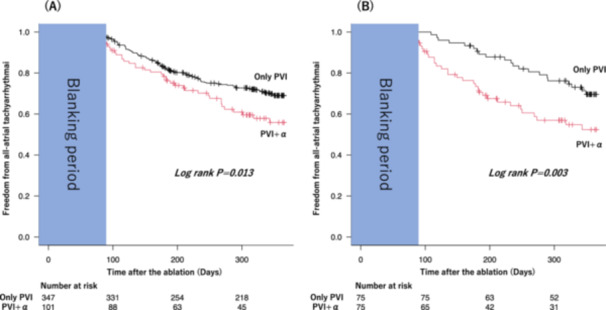
Kaplan‐Meier curve of all atrial tachyarrhythmia‐free survival for (A) all patients and (B) patients who underwent propensity score matched analysis.

The median follow‐up period was 363 days (288–381 days). At 1 year follow‐up, the Kaplan‐Meier curve estimate of any atrial tachyarrhythmia‐free survival indicated significantly fewer recurrences of atrial tachyarrhythmia in the only PVI group (69% vs. 56%; *p* = 0.013, Figure 2A). When focusing solely on the PVI + LAPW isolation, no statistically significant difference was observed in the recurrence rate of any atrial tachyarrhythmia (Supplemental material A). (/R1:R8) In the univariate analysis, extra PV ablation was a significant predictor of atrial tachyarrhythmia recurrence after ablation procedure. (hazard ratio, 2.169 [95% CI, 1.278–3.682], *p* = 0.004). After adjusting for age, gender, BMI, hypertension, diabetes mellites, heart failure, first AF ablation and prior use of class Ⅰ or Ⅲ AAD, extra PV ablation still showed a significant association with the primary outcome. (hazard ratio, 2.520 [95% CI, 1.446–4.392], *p* = 0.001).

Post‐propensity score matching results also indicated a higher incidence of any atrial tachyarrhythmia recurrence in the PVI + *α* group (70% vs. 52%, *p* = 0.003, Figure 2B).

### 3.2 Freedom From AF and AT/AFL at the 1‐Year Follow‐Up

**Figure 3:**
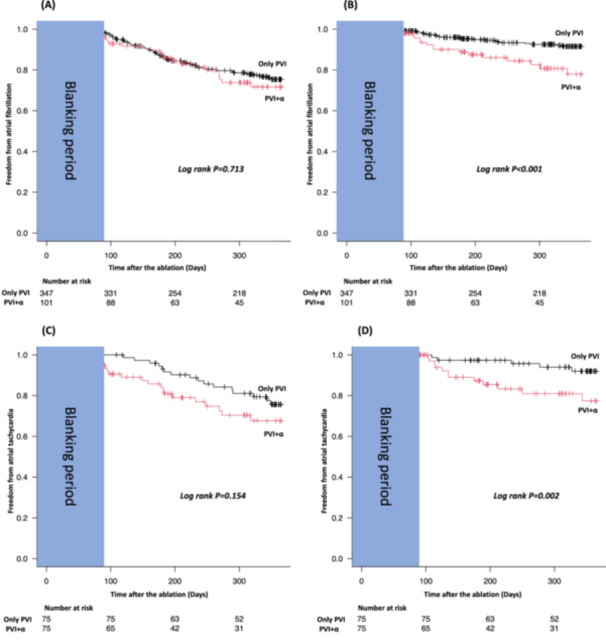
Kaplan‐Meier curve of atrial fibrillation (AF) and atrial tachycardia (AT)/atrial flutter (AFL)‐free survival. (A) Freedom from AF in all patients; (B) Freedom from AT/AFL in all patients; (C) Freedom from AF in patients who underwent propensity score matched analysis; (D) Freedom from AT/AFL in patients who underwent propensity score matched analysis. Abbreviation: AF, atrial fibrillation; AFL, atrial flutter; AT, atrial tachycardia; PVI, pulmonary vein isolation.

Notably, while no significant differences of AF recurrence between the two groups were detected (25% vs. 28%, *p* = 0.713, Figure 3A), AT/AFL recurrence was significantly more common in the PVI + *α* group (8% vs. 22%, *p* < 0.001, Figure 3B). The same result was obtained in the analysis focusing solely on PVI + PWI (Supplemental material B and C). (/R1:R8)

In the propensity score match cohort, AF and AT/AFL recurrence rate after adjustment remained also consistent with the pre‐adjustment analysis (AF recurrence; 24% vs. 32%, *p* = 0.154, Figure 3C/AT/AFL recurrence; 8% vs. 23%, *p* = 0.002, Figure 3D).

### 3.3 Repeat Procedure After an Index PFA Ablation

Out of the 155 patients who had a recurrence of atrial tachyarrhythmia, repeat ablation was performed in 66 patients: 49/113 (43%) in the only PVI group, and 17/42 (40%) in the PVI + *α* group (*p* = 0.855). The indication for the repeat procedure was AT in 11/49 (22%) patients in the only PVI group and in 13/17 (76%) patients in the PVI + *α* group, and recurrent AF in the remaining 38/49 (78%) patients in the only PVI group and in the remaining 4/17 (24%) in the PVI + *α* group (*p* < 0.001). During repeat procedure, 126/191 (66%) of PVs in the only PVI group and 48/67 (72%) of PVs in the PVI + *α* group were found to be durably isolated (*p* = 0.450). Complete durable PVI (i.e. all PVs in an individual patient) was found in 14/49 (29%) patients in the only PVI group and in 8/17 (47%) patients in the PVI + *α* group (*p* = 0.233). Of the 17 patients in the PVI+*α* group, during the index procedure, 14 underwent LAPW isolation, 7 underwent MI ablation, and 1 underwent CTI ablation. Reconduction of the extra PV lesions was observed in 3/14 (21%) for LAPW isolation and 4/7 (57%) for MI ablation. All of these reconducted lesions formed reentry circuits for AT.

### 3.4 Safety Outcomes

The overall incidence rate of complications was 3.6% with 16 events reported in 448 subjects. Procedural major complications were recorded in 7 (2.0%) and 1 (1.0%) in the only PVI and PVI + *α* groups, respectively (*p* = 0.690). Details of complications are summarized in Table 3. There was no statistically significant difference in pericardial tamponade (only PVI vs. PVI + *α*: 6 vs. 0; *p* = 0.345). Of the 6 reported cases, one patient underwent cardiac surgery. All remaining pericardial effusion were drained percutaneously. In addition, stroke was reported in one (0.3%) and one (1.0%) patient, respectively (*p* = 0.400). In total, procedural minor complications were reported in 5 (1.4%) and 3 (3.0%) patients in only PVI and PVI + *α* groups, respectively (*p* = 0.388). Access site complications occurred in 4 (1.2%) patients in the only PVI group, while in 2 (2.0%) patients in the PVI + *α* group (*p* = 0.621). Persistent phrenic nerve palsy and coronary artery spasms were not reported in this trial. In a single patient, a pericarditis unrelated to cardiac tamponade was noted after ablation, which completely recovered after appropriate medical therapy.

**Table 3:** Summary of Complications.

	Entire cohort			Propensity score matched		
	only PVI	PVI + α		only PVI	PVI + α	
	n = 347	n = 101	*p* value	n = 75	n = 75	P value
Major complications, n (%)	7 (2.0)	1 (1.0)	0.690	0 (0)	1 (1.3)	1.000
Pericardial tamponade, n (%)	6 (1.7)	0 (0)	0.345	0 (0)	0 (0)	NA
Stroke, n (%)	1 (0.3)	1 (1.0)	0.400	0 (0)	1 (1.3)	1.000
TIA, n (%)	0 (0)	0 (0)	NA	0 (0)	0 (0)	NA
Minor complications, n (%)	5 (1.4)	3 (3.0)	0.388	0 (0)	2 (2.7)	0.497
Vascular access site complication	4 (1.2)	2 (2.0)	0.621	0 (0)	1 (1.3)	1.000
Prenic nerve dysfunction	0 (0)	0 (0)	NA	0 (0)	0 (0)	NA
Air embolism	0 (0)	1 (1.0)	0.225	0 (0)	1 (1.3)	1.000
Coronary spasm	0 (0)	0 (0)	NA	0 (0)	0 (0)	NA
Haemoptysis	0 (0)	0 (0)	NA	0 (0)	0 (0)	NA
Pericarditis	1 (0.3)	0 (0)	1.000	0 (0)	0 (0)	NA
Pneumonia	0 (0)	0 (0)	NA	0 (0)	0 (0)	NA

Abbreviations: PVI, pulmonary vein isolation; TIA, transient ischemic attack.

## 4 Discussion

The EUropean Real World Outcomes with Pulsed Field AblatiOn in Patients with Symptomatic AtRIAl Fibrillation (EU‐PORIA) registry revealed that the pentaspline PFA catheter was showed to be a safe and effective treatment strategy for AF ablation in a large variant of patients, including paroxysmal and non‐paroxysmal AF patients with an overall atrial tachyarrhythmia recurrence free rate of 74% and a safety event rate of 3.6% [9]. In this trial, we extracted 448 patients with persistent AF from the EU‐PORIA registry and conducted an analysis of the effectiveness and safety of extra PV ablation using a pentaspline PFA catheter.

## 5 Main Findings

The main findings of this trial are as follows:

1. At 1 year follow‐up, the recurrence rate of all‐atrial arrhythmia was significantly higher in the PVI + *α* group even after propensity score match. In more detail, while there was no significant difference in the recurrence rate of AF, there was a higher recurrence rate observed in AT/AFL.
2. During the performance of extra PV ablation, there was a tendency to use a combination of a 3D mapping system, general anesthesia, and the 35 mm ablation device more frequently.
3. The procedural and fluoroscopy times were significantly shorter in the only PVI group.
4. The incidence of procedure‐related complications did not show a significant difference regardless of the performance of extra PV ablation.

### 5.1 Patient Characteristics in Persistent AF

In univariate analysis, the PVI + *α* group exhibited significantly higher rates of diabetes mellites, preoperative AAD usage, and the number of redo procedures. In other words, the PVI + *α* group included a higher proportion of patients who had experienced a recurrence of persistent AF after some form of catheter ablation previously. To adjust potential confounding factors, propensity score matching analysis was performed in this trial. After propensity score matching, there was no significant difference in baseline characteristics.

### 5.2 Clinical Outcomes in Persistent AF After Ablation Using a Pentaspline PFA Catheter

In a few years, PVI using a pentaspline PFA catheter is established in patients with paroxysmal and non‐paroxysmal AF [7, 8, 9, 11, 12, 13, 14]. The pentaspline PFA catheter was developed as one‐shot PVI device, however durable extra PV lesions, such as LAPW ablation and MI ablation, can be also achieved using the configure‐changeable PFA catheter [8]. In this study, patients who underwent extra PV ablation had a higher recurrence of all‐atrial arrhythmias compared to those who underwent PVI alone. Among them, while there was no significant difference in the recurrence of AF, patients who underwent extra PV ablation had a higher recurrence of AT/AFL. In a meta‐analysis regarding LAPW isolation, adjunctive LAPW isolation using either radiofrequency or a cryoballoon reduced AF recurrence in patients with persistent AF [15]. In the multicenter registry, however, additional LAPW isolation using a pentaspline PFA catheter did not improve the recurrence rate of any atrial tachycardia [16, 17]. On the other hand, in this study, the extra PV ablation group had a higher recurrence of atrial tachyarrhythmia. This is thought to be due to the higher recurrence of AT/AFL in the PVI + *α* group. The repeat procedure data revealed that, particularly in MI lesions, reconducted extra PV lesions caused AT in the long term. This may have contributed to the high recurrence of AT in the PVI + *α* group. Additionally, our group previously reported that, in cases of recurrence with AT, there was a higher incidence of recurrence through a roof‐dependent AT involving the narrow zone between the previous PVI lesions [18]. Patients who underwent PVI alone were more likely to use smaller 31 mm catheters (74% vs. 49%, *p* < 0.001). Depending on left atrial anatomy, theoretically, using a larger 35 mm catheter could result in a narrower distance between unintentionally created PVI lesions on the LAPW. Therefore, patients who underwent PVI with a 31 mm catheter may be less prone to recurrence of AT.

A preclinical study demonstrated the marvelous rate (100%) of durable LAPW isolation with invasive remapping study 3 months after procedure. However, chronic CTI block was observed only in 75% patients [8]. Additionally, Davong et al. reported an extremely high success rate in achieving bidirectional block in the MI using a pentaspline PFA catheter [19]. On the other hand, Kueffer et al. reported difficulties in creating a complete bidirectional block in the MI, necessitating touch‐up with an radiofrequency catheter in 50% (3/6 patients) of cases [20]. In our study, among patients who underwent extra PV ablation, 37% underwent MI ablation, and 3% underwent CTI ablation. Compared to LAPW isolation, MI ablation and CTI ablation are challenging in forming durable lesions due to anatomical complexities such as thick tissue in the valve annulus and the presence of the eustachian ridge. Additionally, due to the stunning phenomenon, it can be challenging to determine whether a durable lesion has been created in the acute phase [21, 22]. As a result, the high incidence of AT/AFL recurrence in the PVI + *α* group may be associated with procedural factors, including iatrogenic conduction gaps or incomplete linear blocks [23, 24, 25]. This result was obtained by performing extra PV ablation with the pentaspline PFA catheter, and using the focal PFA catheter for line ablation might reduce the recurrence of AT/AFL. Furthermore, it should be taken into consideration that adding extra PV ablation can result in longer procedural and fluoroscopy times, potentially increasing the burden on both patients and medical staff.

### 5.3 Safety Outcomes in Persistent AF After Ablation Using a Pentaspline PFA Catheter

The overall safety event rate (3.6%) was similar to those previously reported for real‐world experiences with thermal ablation modalities [26, 27, 28]. The overall incidence rate of pericardial tamponade was 1.3%, and there was no significant difference in the incidence rate even with the performance of extra PV ablation. In the EU‐PORIA registry, a significant portion of these events was attributed to the learning curve of the procedure, and the incidence rate was mitigated by improving the procedure workflow [9]. There was no significant difference in the incidence rates of stroke or transient ischemic attack between both groups, and there were no procedure‐related deaths. While MI ablation and CTI ablation were performed in 40% of patients as part of extra PV ablation, coronary artery spasms or significant ST elevation were not reported. Invasive testing revealed coronary artery spasms in 41.2% of patients during MI ablation using a pentaspline PFA catheter, but most of these cases were subclinical [29].

### 5.4 Ablation Strategy Using a Pentaspline PFA Catheter for Persistent AF

This study revealed that the recurrence rate of AF was no difference between the two groups, however, extra PV ablation alongside PVI contributed to high recurrence rate of AT/AFL. On the other hand, the higher recurrence of AT/AFL suggests that these arrhythmias are more likely to be successfully terminated through a redo procedure. (/R1:R9) The use of a pentaspline PFA catheter for extra PV ablation did not alter the safety profile. However, recent multicenter study also revealed that empirically adding LAPW isolation using pentaspline PFA catheter did not improve the recurrence rate of all‐atrial arrhythmias [16, 17]. Thereby, LAPW isolation should not be performed without hesitation unless non‐PV foci in the LAPW or unintentional narrow zones between PVI lesions are created. In our study, PWI was defined as the area between the two lines connecting the superior and inferior PVs, as commonly used with the pentaspline PFA catheter. However, the region below the inferior line, extending to the endocardial aspect of the coronary sinus, is crucial for complete posterior wall isolation. Failure to address this area may leave a substrate for future arrhythmias. While our study did not explore this extension, incorporating it could potentially improve treatment outcomes, particularly in early persistent AF. This warrants further investigation in future studies. (/R1:R4)

For MI ablation and CTI ablation as well, due to the potential risk of incomplete ablation lesions or remote conduction gaps leading to the recurrence of AT/AFL, it is not advisable to perform empiric ablation. The currently available pentaspline PFA catheter does not feature contact force, making it difficult to accurately assess tissue contact even when used in conjunction with a 3D mapping system. Insufficient contact may lead to iatrogenic substrates in the remote period, potentially causing a high recurrence rate of AT/AFL. (/R1:R2) If extra PV ablation using the pentaspline PFA catheter is deemed necessary, real‐time evaluation of catheter contact, such as using intracardiac echocardiography or integrating 3D mapping, may be essential. As we reported in our previous 5S study, the pentaspline PFA device allows for simple and safe simple single shot PVI using standard sedation [12]. In ablation for persistent AF using a pentaspline PFA catheter, a simple and safety PVI strategy is desirable to reduce the recurrence rate of atrial tachyarrhythmia.

### 5.5 Limitation

This study is a retrospective, non‐randomized observational study using the EU‐PORIA registry, which introduces the potential for confounding factors. Particularly, the impact of patient selection is significant since assignment to the only PVI group and PVI + *α* group was not prospectively randomized. For example, the severity of AF/atrial substrates, such as the size of AF and the presence of substrates that can cause organized atrial tachyarrhythmia, may have influenced the results of this study. To address this, Propensity Score Matching was conducted to adjust for patient confounding factors. However, due to the small sample size, not all clinically significant factors were adjusted for in the propensity score match. For the correction of hidden confounding factors, a prospective randomized study would be necessary. This study analyzes only persistent AF and does not include long‐lasting AF (defined as a duration of more than 1 year). (/R1:R1) In patients with long‐lasting AF, a more aggressive approach is probably required. (/R1:R6) Furthermore, ablation workflow and patient management were all performed according to the standard‐of‐care at each center. Thereby, the procedural maneuver was not standardized, making it difficult to properly assess the effectiveness of each extra PV ablation. (/R1:R7) Follow‐up monitoring and data collection were performed based on each center's standard practice. We did not provide mobile ECG devices or perform loop recorder implantation. Therefore, we anticipate the possibility of under‐detection of arrhythmias. Additionally, the inability to analyze AF burden is a major limitation of this study. (/R1:R3) In terms of the procedural aspects, the devices and systems used during ablation are chosen at the discretion of each operator. Decisions regarding the addition of extra PV ablation and the selection of ablation sites are also left to the judgment of each institution. The use of the pentaspline PFA catheter for extra PV ablation is currently considered an off‐label indication.

Systematically collecting real‐world clinical data after the commercialization of a medical device is crucial. The data obtained from this clinical study on the effectiveness and safety of ablation procedures using the pentaspline PFA catheter for persistent AF could serve as a significant indicator in shaping future treatment strategies for persistent AF.

## 6 Conclusion

PVI plus extra PV ablation using a pentaspline PFA catheter is associated with a higher incidence of atrial tachycardia recurrences. In ablation for persistent AF, a simple approach involving PVI alone is considered desirable.

## Conflicts of Interest

**Jun Hirokami:** none. Kyoung Ryul Julian Chun is a consultant for and has received honoraria as well as research funding from Abbott, Medtronic, Boston Scientific, and Biosense Webster. Stefano Bordignon has received honoraria from Medtronic and Biosense Webster. Shota Tohoku: none. Kars Neven is a consultant for Biosense Webster, Boston Scientific, Field Medical, and LifeTech Scientific. Tobias Reichlin has received research grants from the Goldschmidt‐Jacobson Foundation, the Swiss National Science Foundation, the Swiss Heart Foundation, and the sitem insel support fund, all for work outside the submitted study. He has received speaker/consulting honoraria or travel support from Abbott/SJM, Bayer, Biosense‐Webster, Biotronik, Boston‐Scientific, Daiichi Sankyo, Farapulse, Medtronic, and Pfizer‐BMS, all for work outside the submitted study. He has received support for his institution's fellowship program from Abbott/SJM, Biosense‐Webster, Biotronik, Boston‐Scientific, and Medtronic for work outside the submitted study. Yuri Blaauw has received research grants from Medtronic and Atricure. He has received speaker/consulting honoraria from Abbott/SJM and Boston‐Scientific, all for work outside the submitted study. Jim Hansen: speakers fees from Boston Scientific and Biosense Webster. Raquel Adelino: none. Alexandre Ouss: none. Anna Füting: educational grant from Boston Scientific. Laurent Roten received research grants from Medtronic and speaker/consulting honoraria from Abbott and Medtronic. Bart A.Mulder: none. Martin H.Ruwald: none. Roberto Mené: none. Pepijn van der Voort: none. Nico Reinsch: consultant for Boston Scientific. Thomas Kueffer: none. Serge Boveda is consultant for Medtronic, Boston Scientific, Microport, and Zoll. Elizabeth M.Albrecht: salaried employees of Boston Scientific and Boris Schmidt is a consultant for and has received honoraria as well as research funding from Abbott, Medtronic, Boston Scientific, and Biosense Webster. Central illustration 1.

**Central illustration 1:**
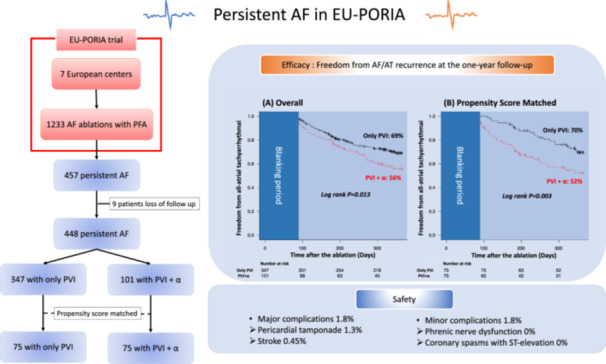
A total of 457 persistent AF patients from EU‐PORIA were included in this trial and Propensity score matched (PSM) analysis was conducted. The Kaplan‐Meier curve of all‐atrial arrhythmia‐free survival for (A) all persistent patients and (B) patients who underwent PSM conducted to the higher recurrence rate in PVI + *α* group.

## Supporting information

Supporting information.

## Data Availability

The data that support the findings of this study are available on request from the corresponding author. The data are not publicly available due to privacy or ethical restrictions.
