# Supplementary material for: Pulsed‐Field Ablation for Persistent Atrial Fibrillation in EU‐PORIA Registry
Source: J Cardiovasc Electrophysiol. 2025 Apr 2;36(8):1710–20. doi: 10.1111/jce.16583 (PMC12337630; doi:10.1111/jce.16583)
Supplement: Supplementary file 1 — Supporting information. [file JCE-36-1710-s001.docx]

1. Survival analysis in patients who underwent posterior wall isolation alongside PVI

Kaplan-Meier curve of atrial fibrillation (AF) and atrial tachycardia (AT)/atrial flutter (AFL)-free survival. (A) Freedom from all atrial tachyarrhythmia (69% versus 58%; P=0.396) ; (B) Freedom from AF (75% versus 74%; P=0.801) ; (C) Freedom from AT/AFL (92% versus 78%; P=0.035)

Abbreviation: AF, atrial fibrillation; AFL, atrial flutter; AT, atrial tachycardia; LAPW, left atrial posterior wall; PVI, pulmonary vein isolation
